# CSACI Position statement on the testing of food-specific IgG

**DOI:** 10.1186/1710-1492-8-12

**Published:** 2012-07-26

**Authors:** Stuart Carr, Edmond Chan, Elana Lavine, William Moote

**Affiliations:** 1 University of Alberta, Department of Pediatrics, Division of Allergy and Immunology, 903 College Plaza 8215-112 Street, Edmonton, AB, T6G 2C8, Canada; 2 BC Children’s Hospital, Department of Pediatrics, Division of Allergy, Room 1C31B – 4480 Oak Street, Vancouver, BC, V6H 3 V4, Canada; 3 Humber River Regional Hospital, Department of Pediatrics, Pediatric Allergy Clinic, Suite 103 – 2115 Finch Ave West, Toronto, ON, M3N 2 V6, Canada; 4 University of Western Ontario, Department of Medicine, Division of Clinical Immunology and Allergy, South Street Campus 2064 – 375 South Street, London, ON, N6A 4 G5, Canada

## 

The Canadian Society of Allergy and Clinical Immunology (CSACI) is very concerned about the increased marketing of food-specific immunoglobulin G (IgG) testing towards the general public over the past few years, supposedly as a simple means by which to identify “food sensitivity”, food intolerance or food allergies. In the past, this unvalidated form of testing was usually offered by alternative or complementary health providers, but has now become more widely available with direct-to-consumer marketing through a nationwide chain of pharmacies.

There is no body of research that supports the use of this test to diagnose adverse reactions to food or to predict future adverse reactions. The literature currently suggests that the presence of specific IgG to food is a marker of exposure and tolerance to food, as seen in those participating in oral immunotherapy studies. Hence, positive test results for food-specific IgG are to be expected in normal, healthy adults and children. Furthermore, the inappropriate use of this test only increases the likelihood of false diagnoses being made, resulting in unnecessary dietary restrictions and decreased quality of life. The immediate expense of the test to individuals (see below for details on cost) will be compounded by the costs incurred by an already-overburdened health care system. Confused by the information provided by IgG testing, individuals are likely to request additional specialist referrals and investigations which would otherwise not be necessary.

Additionally, and perhaps of greater potential concern, a person with a true immunoglobulin E (IgE)-mediated food allergy, who is at significant risk for life-threatening anaphylaxis, may very well not have elevated levels of specific IgG to their particular allergen, and may be inappropriately advised to reintroduce this potentially deadly item into their diet.

As a result of these serious and growing concerns, the CSACI has elected to issue a formal statement supporting the opinions expressed by the American Academy of Allergy Asthma and Immunology (AAAAI) [1], and by the European Academy of Allergy and Clinical Immunology (EAACI) [2]. Both of these organizations warn about the inappropriate measurement of food-specific IgG or IgG4 to suggest the presence or potential of adverse reactions to food. Recent guidelines emphasize that such testing plays no role in the diagnosis of food allergy or intolerance [3]. A recent Canadian publication also elaborates similar concerns from the perspective of community allergy practice [4].

In addition to content of all of the above documents, the CSACI is very concerned about the following issues:

1. The testing process is widely available in Canada, through a variety of complementary health providers, paramedical clinics, and some physicians.

2. A testing kit product is being sold directly to customers, in pharmacies.

3. Marketing strategies for the testing have included the placing of promotional materials in the waiting rooms of physicians without their knowledge or consent.

4. The price of the testing is often in the $400-$700 range, and some third-party payers offer reimbursement despite a clear lack of supporting evidence.

5. The test is also being marketed to concerned parents, and may lead to exclusion diets which carry risks of poor growth and malnutrition for their children: for example, the elimination of dairy products, wheat, eggs, and/or other foods found in healthy balanced diets.

## In summary

The CSACI does not support the decision of licensed physicians and our pharmacist colleagues to offer such testing, given the overwhelming consensus against the validity of such tests.

The CSACI strongly discourages the practice of food-specific IgG testing for the purposes of identifying or predicting adverse reactions to food. We also wish to remind the medical community that blood testing of any kind cannot substitute for consultation with a trained and accredited medical professional such as an Allergist/Immunologist for the diagnosis and management of adverse reactions to food.

## Competing interests

The authors declare that they have no competing interests.

## Authors’ contribution

This Position Statement was the product of an *ad hoc* committee of the Canadian Society of Allergy and Clinical Immunology. Each of the credited authors contributed substantially throughout the planning, drafting and revision stages of the document, and all authors read and approved the final manuscript.
